# Effects of glucose on the uptake and metabolism of glycine in pakchoi (*Brassica chinensis* L.) exposed to various nitrogen sources

**DOI:** 10.1186/s12870-017-1006-6

**Published:** 2017-03-02

**Authors:** Qingxu Ma, Xiaochuang Cao, Yinan Xie, Han Xiao, Xiaoli Tan, Lianghuan Wu

**Affiliations:** 1 Ministry of Education Key Lab of Environmental Remediation and Ecosystem Health, College of Environmental and Resource Sciences, Zhejiang University, Hangzhou, 310058 China; 2 Zhejiang Provincial Key Laboratory of Subtropic Soil and Plant Nutrition, College of Environmental and Resource Sciences, Zhejiang University, Hangzhou, 310058 China; 3 State Key Laboratory of Rice Biology, China National Rice Research Institute, Hangzhou, 310006 China

**Keywords:** Brassica chinensis, Glucose, Glycine, Nitrogen uptake, Rhizodeposition, Soil nutrients

## Abstract

**Background:**

Plants can absorb amino acids as a nitrogen (N) source, and glucose is an important part of root rhizodeposition and the soil sugar pool, which participates in the regulation of plant growth and uptake. In pakchoi, the effect of glucose concentration on the glycine N uptake from a nutrient mixture composed of glycine, ammonium, and nitrate, or from a single N solution of glycine alone was studied using specific substrate ^15^N-labeling and ^15^N-gas chromatography mass spectrometry.

**Results:**

The optimal glucose concentration for plant growth was 4.5 μM or 25 μM when supplied with glycine alone or the N mixture, respectively, and resulted in a >25% increase in seedling biomass. The addition of glucose affected the relative contribution from organic or inorganic sources to overall N uptake. When glucose was added at optimal concentrations, glycine was preferentially used as an N source, while the relative contribution from nitrate was reduced. The limiting step for glycine N contribution was active uptake in the roots in high glucose and single-N-source conditions; however, root metabolism of glycine to serine was limiting in high-glucose and mixed-N-source conditions.

**Conclusions:**

The addition of low concentrations of glucose increased the relative uptake of organic nitrogen and reduced the uptake of nitrate, suggesting a feasible way to decrease nitrate content and increase the edible quality of vegetables.

## Background

Nitrogen (N) is a major component of proteins and is important for plant growth. To date, most studies of N uptake focus on inorganic N sources such as ammonium and nitrate and have shown that the addition of excess inorganic N has significant negative effects on both soil and environmental health [1]. In contrast to inorganic N, organic N sources from amino acids, nucleic acids, quaternary ammonium compounds, and proteins can be directly acquired and metabolised by plants [2–5], bypassing the need for decomposition by microorganisms. Amino acids account for more than 50% of the total N uptake in low-temperature ecosystems including the arctic, alpine tundras, boreal forests, and heathlands [6–9]. Although the actual contribution of amino acids to overall N uptake in natural environments has not been determined due to the lack of an accurate assessment method, organic N, especially amino acids, may be an unrecognised resource for plant N nutrition.

The uptake and metabolism of organic N is affected by several abiotic and biotic factors that include light intensity, pH, soil moisture and texture, and CO_2_ level [10–14]. For example, at 11 °C glycine uptake is limited, while at 21 °C the metabolic conversion of glycine to serine is the limiting step in its N contribution [15]. In a previous study, we showed that higher light intensities increased the uptake of glycine and nitrate, and lower light intensities increased the percentage of ammonium uptake; moreover, we found that the metabolism of ammonium produced by glycine might limit the relative contribution from glycine to the overall N uptake under high light conditions [16]. An in-depth understanding of how environmental factors affect N uptake and metabolism could aid in regulating plant nutrition and the data could be used to predict the future performance of plants in response to global climate change.

Nitrogen uptake and metabolism are closely linked to carbon (C) content, as the carbon skeleton is essential for the assimilation of inorganic N into amino acids, nucleic acids, and proteins [17]. Enhanced supplies of C increase the uptake and metabolism of N [18, 19] because the process of N metabolism requires energy and C skeleton consumption [20]. Multiple studies have shown that N-responsive genes are closely related to C signalling pathways [21, 22], but the mechanisms associated with crosstalk between C and N remain obscure [17]. Therefore, an in-depth knowledge of these interconnections could aid in understanding the regulation of plant nutrition especially N uptake and ultimately apply to crop quality improvement.

The effect of sugars on nitrate uptake is well studied, but no studies have examined the effect of sugars on amino acid uptake and metabolism. Sugars enhance nitrate reductase activity and decrease its degradation [23]. Therefore, changes in the sugar supply can lead to alterations in the expression of numerous genes involved in ammonium metabolism, nitrate uptake, reduction, amino acid synthesis, and protein synthesis [24]. De et al. (2014) suggested that sucrose in roots affects both NO_3_^−^ transporters and assimilatory genes, and showed that glucose stimulates the expression level of the high-affinity transporter *NRT2.1* protein and nitrate transport activity independent of the stimulation of protein expression. It has also been demonstrated that post-transcriptional mechanisms influence NO_3_^−^ uptake in response to sucrose [25]. Remarkably, most experiments have been conducted with nitrate only, and information on the effects sugar has on the relative uptake of different sources of N is limited. It is interesting that amino acids can be taken up by plants directly and that partial replacement of nitrate by amino acids could improve vegetable quality; however, they also reduce biomass compared to nitrate supplementation [26]. The absorption of amino acids by plant roots is associated with two benefits: 1) amino acids are already in the required reduction state for N uptake, which saves the energy required for inorganic N metabolism; and 2) the assimilation of pre-formed carbon skeletons from organic nitrogen sources reduces the biosynthetic costs of plant growth [27]. Consequently, the effects of externally supplied C on amino acid uptake and metabolism may differ with the source of inorganic N and may alter the relative uptake of inorganic and organic forms of N.

Pakchoi (*Brassica chinensis* L.) is cultivated over a large northern to southern range in China. Most soil solutions contain high glycine content [28], and therefore, we selected it as a representative amino acid for testing how glucose affects N uptake in pakchoi. To avoid the decomposition of amino acids by microorganisms, pakchoi seedlings were hydroponically cultivated in a sterilised environment and ^15^N labelling was used to test the following: (1) how glucose affects pakchoi growth and the relative uptake of different N forms; (2) how different glucose concentrations affect the uptake and metabolism of glycine; and (3) whether the effect of glucose on glycine uptake and metabolism was influenced by the N supply.

## Methods

### Seedling culture conditions

Pakchoi plants were cultured in a sterile environment as described [16]. Briefly, pakchoi seeds (provided by Zhejiang Academy of Agricultural Sciences) were soaked in purified water overnight and then sterilised using the method previously described [29]. The seeds were placed into sterilised culture dishes for 3 d with day/night temperatures of 25/20 °C, humidity of 60%/40%, and a 12 h light cycle (360 μmol⋅m^−2^⋅s^−1^). Seedlings were transferred to a 50-mL centrifuge tube containing 0.3% cooled agar dissolved in water and placed in a sterilised culture room under the aforementioned conditions. After 1 day of growth, the seedlings began to grow out of the holes in the tube caps and the holes were then sealed with silicone rubber (Nanda 704, China). The seedling and the tube cap were transferred to a new centrifuge tube that was filled with a nutrient solution. The nutrient solution contained 2 mM K_2_SO_4_, 4 mM CaCl_2_, 1.4 mM MgSO_4_ · 7H_2_O, 2 mM KH_2_PO_4_, 0.1 μM NaMoO_4_ · 2H_2_0, 1 μM ZnSO_4_ · 7H_2_O, 0.4 μM CuSO_4_ · 5H_2_O, 8 μM H_3_BO_3_, 5 μM Na_2_EDTA, 18.3 μM FeSO_4_ · 7H_2_O, and 10 μM MnCl_2_. The pH of the solution was adjusted to 6.2, and the composition of solution were the same in each experiment (except N). The N mixtures used in the different experiments were filter-sterilised through a 0.22-μm membrane filter (Millipore, PES Membrane, Ireland) and added to the nutrient solution before use. The materials and nutrient solution without N were autoclaved at 121 °C for 30 min. The nutrient solutions were changed every 3 d at a clean bench in the sterilised culture room.

### Experiment 1: Effect of glucose concentration on the growth and N uptake in pakchoi

Pakchoi seedlings were pre-cultured as described in 3 mM mixed N (1 mM glycine + 1 mM nitrate + 1 mM ammonium) for 6 d before 48 similarly sized seedlings were selected for experimentation to compensate for the natural growth difference in plant populations. Glucose was then added at concentrations of 0, 1.5, 4.5, 10, 25, 50, 500, and 5,000 μM to the nitrogen-containing nutrient solutions and the mixtures were filter-sterilised. The seedlings were cultured as described for 15 d with sufficient nutrient supply, after which shoots and roots were harvested separately. The roots were washed in 50 mM CaCl_2_ in an ultrasonic bath for 1 min and then washed three more times in purified water. The shoots and roots were freeze-dried (Labconco Freeze System, USA) and ground into a fine powder using a ball mill (Retsch MM301, Germany). The N content was determined in six replicates from each treatment using the Micro-Kjeldahl method.

Seventy-two similar seedlings were pre-cultivated for 15 d, after which the roots and centrifuge tubes were washed with sterilised purified water. The seedlings were cultivated in 1 mM 10.0% ^15^N-glycine with glucose concentrations of 0, 0.5, 1.5, 4.5, 10, 25, 50, and 500 μM for 15 d before the shoots and roots were harvested separately. The highest glucose level (5,000 μM) was not used because it killed the pakchoi seedlings. Three seedlings were pooled to account for variation between plants in each treatment, and each treatment was replicated three times. The roots were washed and the shoots and roots were freeze-dried separately and ground into a powder as described above. The N content and ^15^N incorporation into the samples were determined using an Elemental Analysis-Stable Isotope Mass Spectrometer (IsoPrime100, UK). In addition, three “blank” seedlings were reserved for each treatment by providing unlabelled N at the same composition as the treated plants. This control design was the same in Experiments 2 and 3 as well. The variation in the growth between the different treatments can affect the natural ^15^N abundance in tissues; therefore, we detected N content for each treatment, and three seedlings were pooled and treated as one replicate.

### Experiment 2: Effect of glucose on the N contribution from ammonium, nitrate, and glycine using mixed sources of N

Pakchoi was pre-cultivated as described in Experiment 1 for 6 d, and 81 similar seedlings were selected. The glucose concentrations used were 0, 25 (the optimal concentration for pakchoi growth as shown in Experiment 1), and 500 μM (an excessive concentration for pakchoi growth); and three N mixtures of the same concentration were prepared for each glucose condition (1 mM NO_3_^−^, NH_4_^+^, and glycine) where only one N source was labelled by ^15^N (5.0% ^15^NO_3_^−^, 5.0% ^15^NH_4_^+^, or 5.0% ^15^N-glycine). For example, under 500 μM glucose, the N mixtures were composed of NO_3_^−^:NH_4_^+^:^15^N-glycine, NO_3_^−^:^15^NH_4_^+^:glycine, or ^15^NO_3_^−^:NH_4_^+^:glycine, and the N uptake and contribution from the different N sources could be separated based on this labelling scheme. The nine treatments (3 N sources × 3 glucose concentrations) were replicated three times, the cultivation solution was changed every 3 d, and the plants were harvested at 21 d. The shoots and roots were harvested separately and three seedlings were pooled into a single sample. The samples were washed, dried, ground, and the ^15^N content was detected as described in Experiment 1.

### Experiment 3: Effect of glucose on the short-term uptake of glycine

Pakchoi seedlings were pre-cultivated for 25 d and 180 similar seedlings were selected. Seedlings were “starved” overnight in an N-free nutrient solution before beginning the short-term (4 h) glycine uptake tests. The glucose concentration in this experiment was determined based on the N source. The optimal glucose concentration for pakchoi growth was 4.5 μM when a single N source was used and 25 μM under the mixed nitrogen conditions (Experiment 1). Therefore, 45 seedlings were cultivated in 1 mM 98.10% ^15^N-glycine for 4 h at glucose concentrations of 0, 4.5, and 500 μM; and an additional 45 seedlings were cultivated for 4 h with 1 mM NO_3_^−^ + 1 mM NH_4_^+^ + 1 mM 98.10^−15^N-glycine at glucose concentrations of 0, 25, and 500 μM.

Simultaneously, the protonophore carbonyl cyanide m-chlorophenylhydrazone (CCCP) [30], that inhibits the active uptake of glycine was used to examine the effect of glucose on the active and passive uptake of glycine. Ninety seedlings were starved overnight in an N-free nutrient solution and pre-treated with 50 μM CCCP for 1 h, after which 45 seedlings were cultivated for 4 h with either 1 mM 98.10%-^15^N-labelled glycine in glucose concentrations of 0, 4.5, and 500 μM, or 1 mM NO_3_^−^ + 1 mM NH_4_^+^ + 1 mM 98.10 ^15^N-glycine in 0, 25, and 500 μM glucose. The uptake tests for CCCP-treated and untreated specimens were conducted simultaneously with three replicates per treatment. The roots and shoots were harvested separately and five seedlings were pooled as one sample for each experimental replicate. The roots were washed, dried, and analysed as described in Experiment 1. The ^15^N values obtained from CCCP-treated samples represented the passive uptake of glycine in pakchoi.

### Experiment 4: Effect of Glucose on N metabolic enzyme activity

After root N uptake, several enzymes metabolise glycine; is the metabolism of glycine changed by the uptake of glycine? Ninety-six seedlings were pre-cultured for 22 d and then starved overnight as previously described in Experiment 3. Forty-eight seedlings were cultivated for 4 d in 1 mM glycine in glucose concentrations of 0, 4.5, and 500 μM. The remaining 48 seedlings were cultivated in glucose concentrations of 0, 25, and 500 μM added to a mixed N source solution of 1 mM NO_3_^−^ + 1 mM NH_4_^+^ + 1 mM glycine. The nutrient solution was changed every 2 d. The seedlings were harvested and six seedlings were pooled to represent one sample in each of the four experimental replicates. The activities of glutamine synthetase (GS) [31], glutamic-pyruvic transaminase (GPT), and glutamic oxaloacetic transaminase (GOT) [32] were measured in roots and shoots.

### Experiment 5: Effect of glucose on amino acid content of pakchoi

Pakchoi seedlings were pre-cultured for 25 d and the 72 uniform seedlings were washed and N-starved overnight. They were then cultivated in 1 mM NO_3_^−^ + 1 mM NH_4_^+^ + 1 mM glycine with 0, 25, and 500 μM glucose for 12 h (25 °C, 60% humidity, 360 μmol⋅m^−2^⋅s^−1^ light). The shoots and roots were harvested separately and the roots were washed as described in Experiment 1. The experiment included six replicates with four seedlings in each replicate to reduce individual plant variation. Aliquots of 1 g fresh shoots and roots were ground in 4 ml of 5% sulfosalicylic acid and incubated for 1 h. The extracts were centrifuged at 14 000 × *g* for 10 min and the supernatant was passed through a 0.22-μm membrane filter (Millipore, PES Membrane, Ireland). Amino acid content was measured with an automatic amino-acid analyzer (L-8900, Hitachi, Japan).

### Experiment 6: Effect of glucose on the metabolism of glycine

Pakchoi seedlings were pre-cultured for 25 d and 96 similar seedlings were selected for further cultivation. The roots were washed several times with purified water and the seedlings were N-starved for 12 h. Then, the seedlings were cultivated in 1 mM NO_3_^−^ + 1 mM NH_4_^+^ + 1 mM 98.1% ^15^N-glycine with 25 μM and 500 μM glucose for 12 h (25 °C, 60% humidity, 360 μmol⋅m^−2^⋅s^−1^ light). The pakchoi roots and shoots were harvested separately; eight seedlings per treatment were pooled and each treatment had 6 replicates. The roots were washed, dried, and ball milled as previously described. The ^15^N-labelled amino acids were detected by gas chromatography–mass spectrometry (GC-MS) as previously described [15] with minor modifications. Briefly, 20 mg aliquots of ball milled root and shoot samples were extracted in 3 ml 80% ethanol for 1 h with gentle shaking every 10 min. The extracted solutions were centrifuged at 3,500 × *g* for 15 min and the supernatant was collected. The extraction and centrifugation of these samples was repeated once more. Each supernatant from the two extractions were combined and dried in a rotary evaporator (EYELA, SB-1100) at 25 °C and resuspended in 1 ml of 0.1 M hydrochloric acid. This solution was centrifuged at 12,000 × *g* for 15 min and the supernatant was added to the Dowex 50WX8-200 cation exchange columns (Sigma-Aldrich, St Louis, MO, USA) (2 ml bed volume, H^+^ form). The cation exchange columns were washed with 20 ml ultrapure water, and 20 ml of 4 M ammonia solution was used to wash out the amino acids. The eluate was blown for 8 h with N_2_ to remove the NH_3_, and then freeze-dried (Labconco Freezen System, U.S.A.). Amino acids in the resultant extracts were derivatised to t-butyldimethylsilyl in 10 μL N-methyl-N-tert-butyldimethylsilyl-trifluoroacetamide. Finally, the ^15^N-labelled amino acids in the roots and shoots were detected by GC-MS.

### Calculations

The uptake of N from different sources was determined based on the ^15^N concentration in treated seedlings relative to that in “blank” seedlings that were not exposed to labeled N. The amounts of NO_3_^−^, glycine, and NH_4_^+^ taken up from the labeled N were calculated using the following equation [33]:

1 $$ {N}_{uptake}={N}_{Total- N}\frac{A_s-{A}_c}{A_f} $$

where *N*_*uptake*_ is the amount of a given N source taken up in the shoots or roots of pakchoi seedlings; *N*_*Total-N*_ is the total N content of the roots or shoots; *A*_*s*_ is the ^15^N atom % in the roots or shoots; *A*_*c*_ is the ^15^N atom % in the “blank” seedlings that were supplied with unlabeled N; *A*_*f*_ is the ^15^N atom % of the labeled-N source (10.0% glycine for Experiment 1; 5.0% glycine, 5.0% NO_3_^−^, or 5.0% NH_4_^+^ for Experiment 2; and 98.1% glycine for Experiment 3, 6).

The proportion of total N taken up from different sources was calculated using the following equation:

2 $$ {N}_{contribution}=\frac{N_{uptake}}{N_{total\hbox{-} N}}\times 100 $$

where *N*_*contribution*_ is the proportion of total N taken up as glycine, NO_3_^−^, or NH_4_^+^ by whole pakchoi seedlings; *N*_*uptake*_ is the amount of a given N source taken up into the roots or shoots, as calculated from equation (1); and *N*_*total-N*_ is the total N total mass of N contained in pakchoi seedlings.

### Statistical analysis

Data are presented as the mean ± standard error (SE). We applied one-way analysis of variance (ANOVA) followed by Duncan’s multiple range method (*p* < 0.05) to assess differences between treatments. All statistical analyses were performed using SAS 8.2 (SAS Institute Inc., Cary, NC). Figures were created using Origin 8.1 (OriginLab, Northampton, MA).

## Results

### Low concentrations of glucose preferentially increase long-term N uptake and pakchoi biomass

**Fig. 1:**
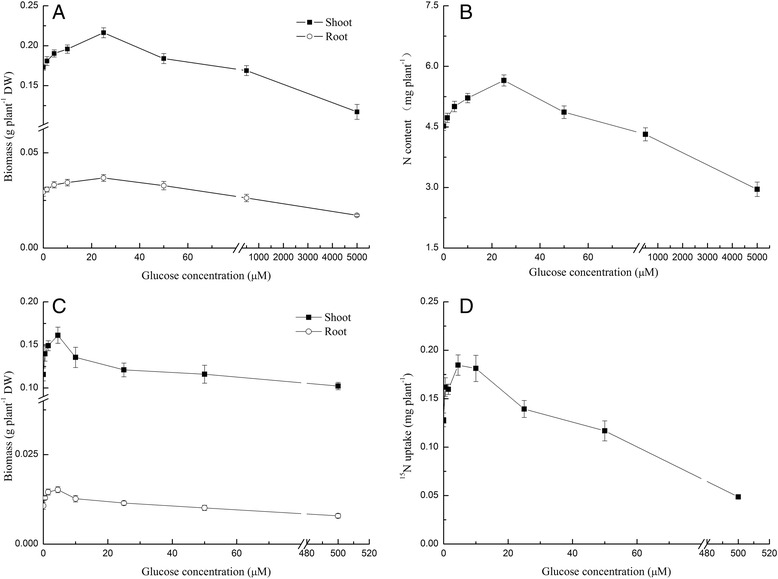
Pakchoi biomass and N uptake under different glucose concentrations. **a** Shoot and root biomass under mixed N source conditions (*n* = 6); **b** N content under mixed N source conditions (*n* = 6); **c** shoot and root biomass under single N source conditions (*n* = 3); **d** ^15^N-glycine uptake under single N source conditions (*n* = 3). Bars indicate mean values ± SE

The growth of pakchoi shoots and roots and the uptake of N increased in ≤ 50 μM glucose concentrations, but were reduced in glucose concentrations >500 μM. In mixed N-source conditions, the optimal glucose concentration that supports pakchoi growth and N uptake was 25 μM (Fig. 1a, b), resulting in a 25% increase in biomass compared to growth in the absence of glucose. When glycine was supplied as the sole source of N, the optimal glucose concentration for pakchoi growth and ^15^N-glycine uptake was 4.5 μM (Fig. 1c, d), which was lower than that observed under mixed N source conditions. These results indicate a preferential increase in pakchoi N-assimilation and growth in the presence of low levels of glucose.

### Glucose preferentially enhances glycine-derived N uptake from mixed N sources

**Fig. 2:**
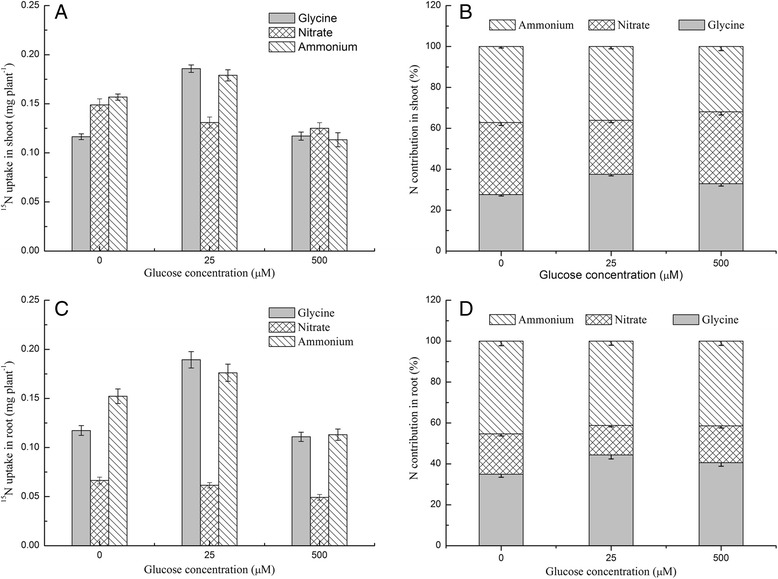
Effects of glucose on ^15^N uptake under mixed N source conditions. The uptake of glycine, nitrate, and ammonia in (**a**) shoots and (**c**) roots. The N contribution of each form of N to the total N uptake (%) in (**b**) shoots and (**d**) roots. Bars indicate mean values ± SE; *n* = 3

Experiment 1 showed that glucose had a significant effect on the growth and N uptake in pakchoi, but how do different levels of glucose affect the uptake of glycine, nitrate, and ammonium? Externally supplied glucose had a significant effect on the relative uptake of N from mixed N sources (Fig. 2). For example, the uptake of ^15^N-glycine and the relative contribution of glycine to overall N uptake when plants were supplied with 25 μM glucose were significantly higher in shoots and roots than in plants grown in the control (0 μM glucose) and high glucose concentrations (500 μM). The uptake and contribution of ^15^N-ammonium to the overall N uptake was highest in the absence of glucose. These results are evidence that glucose can alter N uptake from preferred nitrogen sources, providing a potential tool for supporting amino acid supplementation in pakchoi cultivation. Furthermore, the N contribution from glycine in the shoots and roots of plants grown in the three glucose levels was 27.6–37.5% and 35.0–44.3%, respectively, while the N contribution from nitrate in shoots and roots was 26.4–35.3% and 14.4–19.8%, respectively.

### Glucose concentrations affect the active short-term uptake and transport of glycine

**Fig. 3:**
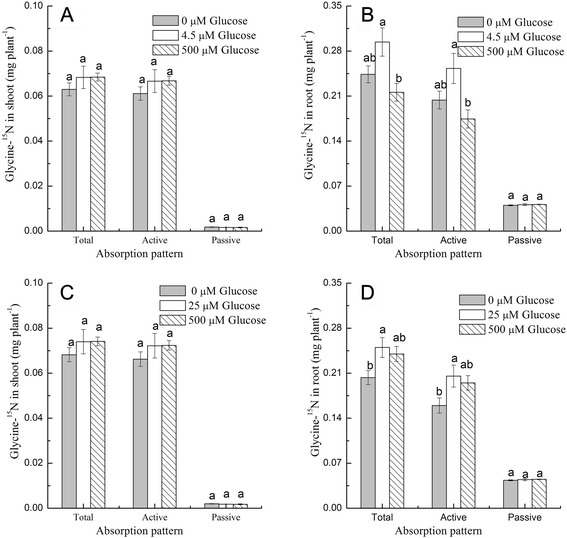
Effects of glucose on the short-term uptake of glycine-^15^N. The Glycine-^15^N uptake was measured in **a** shoots and **b** roots in single N (glycine) source conditions, and in **c** shoots and **d** roots in mixed N conditions. Bars indicate mean values ± SE; *n* = 3. Different letters indicate significant differences between glucose levels (*p* < 0.05)

Glucose changed the uptake and N contribution of glycine whether in single or mixed N sources (Experiment 1 and 2), but was this great difference was caused by root uptake? In roots, glucose had a significant effect on only the active uptake of glycine under both mixed N and single N source conditions (Fig. 3). Under single N source conditions, the active uptake of ^15^N-glycine in the roots at the optimal glucose concentration (4.5 μM) was 29.0% and 45.0% higher than the control (0 μM) and high glucose (500 μM) concentrations, respectively. In mixed N source conditions, the active uptake of ^15^N-glycine in the roots at 25 μM glucose was much higher than that observed in the absence of glucose; however, a similar amount of ^15^N-glycine was detected between the optimal and high glucose treatments in the mixed N conditions. Moreover, in mixed N source conditions, the uptake of ^15^N-glycine with the high glucose concentration was 22.0% higher than the control, which was significantly different from that observed under the single N conditions.

### Glucose concentration can alter the activity of glycine metabolic enzymes in mixed-N conditions

Under mixed N source conditions, the N contribution of glycine decreased under high glucose level in the long-term N uptake test (Experiment 2), but the glycine short-term N uptake amount in high glucose conditions was similar with the optimal glucose level, which prompts the question of whether glycine metabolism inhibits the N contribution of glycine rather than root uptake under high glucose level. Using glycine as a single N source identified no significant differences in the activities of GPT, GOT, and GS in the roots between plants that received the optimal or high glucose treatments (Table 1). However, when supplied with mixed N sources, the GS activity in the shoots and roots were significantly lower at high glucose concentrations than at the optimal glucose concentrations.

**Table 1:** Effects of glucose on the activity of N metabolic enzymes in pakchoi

Glucose concentration (μM)	GPT (μmol · g-1 · 30 min)		GOT (μmol · g-1 · 30 min)		GS (A · mg-1 protein · h-1)	
	shoot	root	shoot	root	shoot	root
0	3.8 ± 0.4a	7.8 ± 1.5a	9.8 ± 0.2ab	12.5 ± 0.2a	19.1 ± 0.7a	23.5 ± 0.6a
4.5	5.7 ± 1.3a	5.7 ± 0.8ab	10.3 ± 0.1a	11.9 ± 0.5a	19.9 ± 0.2a	24.5 ± 2.9a
500	5.9 ± 0.6a	3.9 ± 0.5b	9.6 ± 0.2b	11.2 ± 0.3a	21.3 ± 1.8a	23.4 ± 0.9a
0	4.5 ± 0.9a	5.2 ± 2.7a	12.1 ± 0.2a	13.8 ± 0.4a	19.2 ± 0.4b	24.7 ± 1.0a
25	5.1 ± 0.5a	6.3 ± 1.8a	10.5 ± 0.2b	13.2 ± 0.3a	23.0 ± 0.8a	24.0 ± 0.9a
500	5.9 ± 1.2a	7.2 ± 0.8a	10.2 ± 0.1b	13.1 ± 0.1a	20.7 ± 0.5b	20.5 ± 0.5b

Values represent the mean ± SE (*n* = 3). Different letters in each column indicate significant differences between treatments at *p* < 0.05. The first three rows represent the enzymes under conditions where glycine was supplied as a single N source, and the last three rows represent the enzymes under conditions where glycine was supplied in a mixed N source

### Amino acid content in pakchoi is altered by external glucose concentrations

**Fig. 4:**
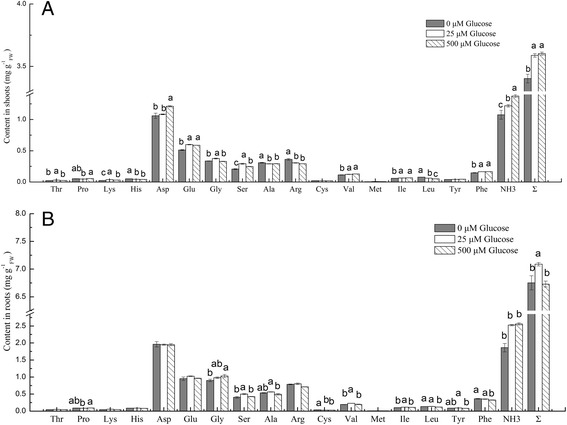
Effects of glucose on amino acids and ammonia contents in pakchoi shoots **a** and roots **b** after a 12-h treatment with 1 mM NO_3_^−^ + 1 mM NH_4_^+^ + 1 mM glycine. Bars indicate mean values ± SE; *n* = 6. Different letters indicate significant differences between glucose levels (*p* < 0.05)

The content of amino acids and ammonia in pakchoi were significantly affected by a 12 h glucose treatment (Fig. 4). Compared to plants with no glucose supplementation, the content of NH_3_ and total amino acids (Σ) in shoots and roots were significantly lower than those in the optimal glucose treatment (25 μM). While the content of total amino acids in shoots showed little difference in optimal and high glucose treatment levels, it was significantly higher in roots of plants that were supplemented with the optimal glucose concentration compared to that of the high glucose treatment. In addition, the content of glycine in roots treated with the high glucose concentration was much higher, and the content of serine was significantly lower than that of plants treated with the optimal glucose concentration, respectively.

### ^15^N-labelled amino acid levels in shoots and roots are affected by glucose supplementation

**Fig. 5:**
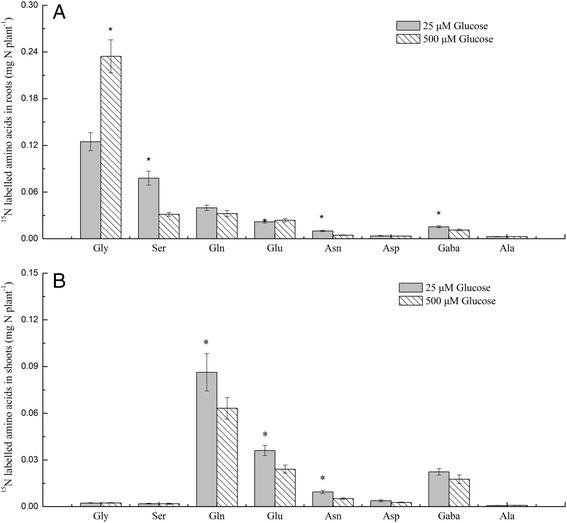
Effects of glucose on the content of ^15^N-labelled amino acids in pakchoi roots (**a**) and shoots (**b**) after a 12-h treatment with 1 mM NO_3_^−^ + 1 mM NH_4_^+^ + 1 mM 98.1% ^15^N-glycine. Bars indicate mean values ± SE; *n* = 6; asterisks indicate significant differences between glucose levels (*p* < 0.05)

Experiment 5 shows that glycine root content in high glucose was higher than in the glucose optimal level; serine was much lower under the same glucose conditions, indicating that the metabolism of glycine compared to serine inhibited the N contribution of glycine at high glucose levels. However, the data collected by the amino-acid analyser has two disadvantages: 1) the amino-acid analyser cannot detect asparagine and glutamine, which are important in glycine metabolism; and 2) whether the detected amino acids are from root uptake of glycine or from the metabolism of nitrate and ammonium cannot be determined. To explore the effect of high glucose levels on the metabolism of glycine, ^15^N labelling and GC-MS were used to detect the ^15^N-labelled amino acids in the pakchoi shoots and roots. Glucose treatment had a significant effect on the ^15^N-labelled amino acid content in pakchoi (Fig. 5). The ^15^N-amino acids varied greatly between shoots and roots. In roots the main ^15^N-labelled amino acids were glycine, serine, glutamine, and glutamic acid; in shoots, the major labelled amino acids were glutamine, glutamic acid, asparagine, and gamma-aminobutyric acid. In roots, ^15^N-glycine was significantly higher in plants cultivated in the high glucose treatment, while serine, gamma-aminobutyric acid, and asparagine levels were significantly lower than those measured in plants treated with the optimal glucose concentration (Fig. 5a). In shoots, asparagine, glutamic acid, and glutamine were significantly higher in the optimal glucose treatment than in the high glucose concentrations (Fig. 5b).

## Discussion

### Effects of glucose on pakchoi growth and N uptake depend on the N conditions

The results of this study indicate that relatively low concentrations of glucose accelerated pakchoi growth, while excessive glucose levels retarded both growth and N accumulation (Fig. 1). A previous study showed that high glucose in plants reduced the rates of photosynthesis and sugar transport, and low glucose in plants led to increased sugar transport activities [34]. In our study, we found that glucose levels alter N uptake, indicating that its regulation is another example of how glucose affects pakchoi growth. The optimal glucose concentration for pakchoi grown in a mixed N source conditions was 25 μM, which is more than five times higher than that observed in single N conditions. This result indicates that the effect of glucose on pakchoi growth is related to the N supply. Similarly, nitrate levels affected the glucose sensitivity of wild-type Arabidopsis during germination [35]. There is a growth inhibition effect at 3% (166 mM) glucose with only 0.1 mM nitrate, but this inhibitory effect was not observed at 5% (275 mM) glucose with either 1 or 5 mM nitrate. In addition, optimal glucose concentrations were found to be 1–3% (55–166 mM) in *Arabidopsis thaliana* seedlings cultivated on agar media [36], which is hundreds of times higher than the optimal concentrations (4.5 or 25 μM) we detected in pakchoi. This large discrepancy may be caused by differences between the plant species and the glucose availability dissolved in water versus that in agar.

Plants possess the ability to take up sugars, which can regulate plant growth and N uptake. Massive amounts of sugar were detected in the xylem sap, which indicated that the glucose in roots might be acropetally transported to the shoots and leaves [37, 38]. Glucose is not only regarded as an energy and carbon resource for biomass production, but it also acts as a rapid molecular signal to coordinate root and shoot development [37]. We show that the optimal concentrations of glucose (4.5 or 25 μM) for pakchoi growth were very low and therefore, might play little role in providing energy and C skeletons for biosynthesis, which suggests that the positive effect of lower glucose concentrations on pakchoi growth results from its role as a signal molecule.

### Effects of glucose on the relative uptake of glycine, nitrate, and ammonium

Several studies have shown that biotic and abiotic factors affect the uptake and metabolism of organic and inorganic N. The optimal glucose concentration increased the relative N contribution from glycine; however, it decreased the N contribution from nitrate and both the optimal and high glucose concentrations decreased the N contribution from ammonium (Fig. 2). Nitrate taken up by roots is transferred to ammonium and assimilated into glutamine by GS, which needs C skeletons for amino acid assimilation [39, 40]. Compared to amino acids, externally supplied glucose may theoretically have a significantly positive effect on the uptake of inorganic N due to the high C demand of inorganic N. However, the opposite effect was observed, as the uptake and N contribution from nitrate in mixed N source conditions was lower at optimal and high glucose concentrations than in glucose-free conditions (Fig. 2b, d). These results could be explained in two ways: first, most studies were conducted under single N source conditions, which neglects to consider the effect of other N sources on the uptake of nitrate. In other words, the uptake of one form of N has an effect on the uptake of other forms [15, 41]. For example, ammonium hinders the uptake of nitrate and the addition of amino acids inhibits the uptake of both ammonium and nitrate [42]. Therefore, the increased uptake of glycine could inhibit the uptake of ammonium and nitrate, as we observed in pakchoi. Second, the optimal glucose concentrations (4.5–25 μM) are very low compared to the concentration of sugars in the plant itself [37, 38], suggesting that sugar may be involved in nutrient signalling and regulation, rather than act as C source.

Significant differences were detected between the relative N contributions from nitrate and glycine in shoots and roots (Fig. 2b, d). Nitrate contributed 26.4–35.3% of total N in shoots, while it accounted for only 14.4–19.8% in roots. By contrast, glycine accounted for 27.6–37.5% and 35.0–44.3% in shoots and roots, respectively. The N contribution of nitrate in shoots was much higher than it in roots, while the contribution of glycine in shoots was much lower than it in roots. This difference likely resulted from variations in both N transportation and metabolism in these two tissues. Most nitrate taken up by roots is transported to and metabolized in shoots [40], which leads to relatively high N levels in shoots. Plants can take up amino acids at a faster rate than nitrate [15, 43, 44]; however, most amino acids are metabolized in the roots [45] and amino acids are allocated to shoots at a slower rate than nitrate [43]. Overall, the differences in the metabolism of glycine and nitrate resulted in significant differences in their relative N contributions.

The objective of reducing nitrate content in vegetables is important because of its potential impact on human health. For example, 80% of human dietary intake of nitrate comes from vegetables [46] and the consumption of leafy vegetables can lead to excessive nitrate ingestion [47]. Excessive amounts of ingested nitrate could be converted to nitrite, which can result in serious potential threats to human health, including the accumulation of carcinogenic nitrosamines or methemoglobinemia [48]. The partial replacement of nitrate with amino acids can be an effective and feasible way to reduce the nitrate content in crop plants. By doing so, plant quality can be enhanced to improve the content of soluble sugar, soluble protein, and free amino acids; however, the biomass of vegetables may also be reduced under these nutrient conditions [49, 50]. In contrast, the present study demonstrates that a higher biomass can be achieved with the addition of glucose at concentrations of 25 μM when compared to the control treatment, and the uptake rates of glycine increase under these conditions, while nitrate uptake decreases. These results suggest that cultivation at the optimal glucose concentration might aid in the reduction of nitrate uptake over other organic N sources, which could ultimately improve vegetable quality. Moreover, studies have shown that most plants possess the ability to utilise amino acids as an N source, but the N contribution from glycine in the sterilised environment may have been overestimated with respect to that in the natural environment. Our results clearly show that pakchoi possesses a strong ability to absorb amino acids, and that glucose plays an important role in this N uptake. Therefore, the results of this study provide a new way to reduce the nitrate content in vegetables via the addition of amino acids in combination with small amounts of glucose to their cultivation conditions.

### Effects of glucose on the uptake and metabolism of glycine

Environmental factors control plant growth by affecting the levels of nutrient uptake, metabolism, transport, storage, and reallocation [51]. Studies of signalling pathways and bottlenecks in amino acid metabolism in plants grown in different environments could help improve the efficiency of N usage [52].

Externally supplied glucose enhanced glycine N contribution by increasing its active uptake and had little effect on its passive uptake. The optimal concentration of glucose increased the active uptake of glycine whether it was included in a single N solution of glycine or in an N mixture (Fig. 3b, e). Most amino acid uptake in plant roots is driven by H^+^-ATPases [53]. This is consistent with our results that show glycine uptake in CCCP un-treated plants was 5.0–7.5 fold greater than that of CCCP-treated plants. Externally supplied glucose might improve active glycine uptake by providing energy or regulating the active uptake. Further research is needed to better describe how glucose is involved in glycine uptake mechanisms in plants.

The mechanism of high glucose inhibited glycine N contribution depending on the N supply. I In the single N solutions composed of glycine alone, active glycine uptake was the limiting step in high glucose concentrations (Fig. 3). Glycine uptake was much lower when glucose was supplied at 500 μM compared to the optimal 25 μM concentration during long-term N uptake tests. In the short-term N uptake tests, the active uptake of glycine was much lower in the high glucose concentration than the optimal concentration, indicating that the active N uptake limiting factor is associated with lower N contribution. Moreover, the N metabolic enzyme activities did not differ significantly between the optimal and high glucose concentrations, suggesting that active uptake is the limiting step rather than the subsequent nitrogen metabolism (Table 1).

**Fig. 6:**
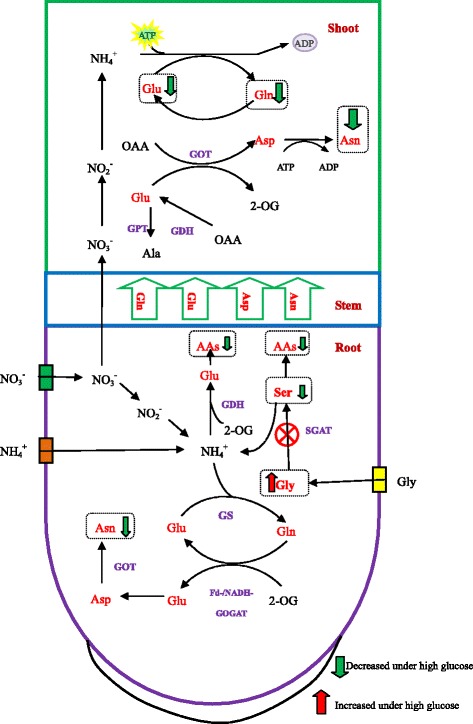
A simplified model of the effect of high glucose on glycine (Gly) metabolism in root and shoot. Gly is transported in root by amino acid transporters, and it is converted to serine (Ser) catalysed by SGAT. Ser can be converted to other amino acids (AAs), and NH_4_^+^ can be converted to glutamine (Gln) catalysed by glutamine synthetase (GS). In addition, Gln with 2-oxoglutarate (2-OG) can be converted to glutamate (Glu), which is catalysed by glutamine synthetase-glutamate synthase (GOGAT). Glu can be converted to aspartic acid (Asp) catalysed by glutamic oxalacetic transaminase (GOT), and Asp can be assimilated into asparagine (Asn). Additionally, NH_4_^+^ can be assimilated to Glu by glutamate dehydrogenase (GDH) and Glu can be converted to alanine (Ala) by glutamic pyruvic transaminase (GPT). ^15^N-labelled Gly was rarely detected in the shoot, indicating that little glycine was transported to shoot. Gln, Glu, Asn, and Asp are the four main amino acids transported from root to shoot, of which Gln is the most important. AA metabolism in the shoot is similar to that in the root. In the root, Gly is high at high glucose, whereas the levels of Ser and Asn are lower compared with the optimal level, indicating that the conversion of Gly to Ser under high glucose level limits Gly metabolism in the root. In the shoot, Asn, Gln, and Glu are low at high glucose, which may be attributable to poor metabolism in root

Under mixed N conditions, the metabolism of glycine to serine was the limiting step for glycine N contribution. The rate of glycine uptake at the high glucose concentration was much lower than that at 25 μM glucose in the long-term test; however, in the short-term test, the uptake of glycine at 500 μM glucose was similar with it at 25 μM glucose, showing that root N uptake was not the limiting step for glycine-N contribution in the mixed N conditions. After root uptake, glycine is converted to serine by serine glyoxalate aminotransferase (SGAT), serine can then be converted to other amino acids and ammonium can be assimilated into glutamine catalysed by GS [40]. Glutamine, along with 2-oxoglutarate, can then be converted to glutamate, which is catalysed by glutamate synthase-glutamine oxoglutarate aminotransferase (GS-GOGAT). Glutamate can be further converted to aspartic acid catalysed by GOT, or converted to be alanine catalysed by GPT [40] (Fig. 6). In pakchoi roots, higher ^15^N-glycine was detected by GC-MS, while little serine, asparagine, gamma-aminobutyric acid, and glutamine was measured under high glucose, indicating that the metabolic process of glycine to serine was inhibited under high glucose. In pakchoi shoots, little ^15^N-glycine was measured, and asparagine, gamma-aminobutyric acid, glutamate, and glutamine were the main ^15^N-labelled amino acids. This is consistent with a previous study that showed that glycine was metabolised by deamination in roots and U-^13^C; ^15^N-glycine was not detected in xylem sap [45]. Furthermore, aspartic acid, asparagine, glutamic acid, and glutamine are the main amino acids transported to the shoots [40]; therefore, root metabolism of glycine to serine is thought to be the limiting step for glycine uptake under high glucose.

Plants transport 20–50% of shoot-fixed C to the roots [54, 55] and some of it is released to the soil as rhizodeposits [54, 56]. Furthermore, root rhizodeposition is regulated by several innate factors and environmental factors, such as root system architecture [57], soil type [58], and nutrient availability [59]. Glucose is one of the largest components of rhizodeposition and the soil sugar pool, which changes constantly. We have shown that small amounts of exogenous glucose can greatly change N uptake and metabolism, which may play an important role in regulating plant responses to biotic and abiotic stress. In addition, high concentrations and availability of amino acids were detected in the rhizosphere [60]; thus, glucose may play an important role in regulating amino acid uptake and metabolism in the soil.

## Conclusions

When supplied with glycine alone or the N mixture, the optimal glucose concentration for plant growth was 4.5 μM or 25 μM respectively, and resulted in the significantly increase in pakchoi biomass. Glycine was preferentially used as an N source when glucose was added at optimal concentrations, while the relative contribution from nitrate was reduced. In high glucose and single-N-source conditions, the limiting step for glycine N contribution was active uptake in pakchoi roots, while root metabolism of glycine to serine was limiting in high-glucose and mixed-N-source conditions. The addition of low concentrations of glucose increased the relative uptake of glycine and reduced the uptake of nitrate, which providing a feasible way to reduce nitrate content and increase the edible quality of vegetables.

## Abbreviations

2-OG: 2-Oxoglutarate
AAs: Amino acids
Ala: Alanine
Asn: Asparagine
CCCP: Protonophore carbonyl cyanide M-chlorophenylhydrazone
GC-MS: Gas chromatography–mass spectrometry
GDH: Glutamate dehydrogenase
Gln: Glutamine
Glu: Glutamate
Gly: Glycine
GOGAT: Glutamine oxoglutarate aminotransferase
GOT: Glutamic oxaloacetic transaminase
GPT: Glutamic-pyruvic transaminase
GS: Glutamine synthetase
N: Nitrogen
Ser: Serine
SGAT: Serine glyoxalate aminotransferase
